# Profiling mitochondrial DNA mutations in tumors and circulating extracellular vesicles of triple‐negative breast cancer patients for potential biomarker development

**DOI:** 10.1096/fba.2023-00070

**Published:** 2023-09-08

**Authors:** Kunwar Somesh Vikramdeo, Shashi Anand, Sarabjeet Kour Sudan, Paramahansa Pramanik, Seema Singh, Andrew K. Godwin, Ajay Pratap Singh, Santanu Dasgupta

**Affiliations:** ^1^ Mitchell Cancer Institute, University of South Alabama Mobile Alabama USA; ^2^ Department of Pathology, College of Medicine University of South Alabama Mobile Alabama USA; ^3^ Department of Mathematics and Statistics University of South Alabama Mobile Alabama USA; ^4^ Department of Biochemistry and Molecular Biology University of South Alabama Mobile Alabama USA; ^5^ Department of Pathology and Laboratory Medicine University of Kansas Medical Center Kansas City Kansas USA; ^6^ The University of Kansas Cancer Center, University of Kansas Medical Center Kansas City Kansas USA; ^7^ Kansas Institute for Precision Medicine, University of Kansas Medical Center Kansas City Kansas USA

**Keywords:** extracellular vesicles, liquid biomarker, mitochondrial DNA, mutation, triple‐negative breast cancer

## Abstract

Early detection and recurrence prediction are challenging in triple‐negative breast cancer (TNBC) patients. We aimed to develop mitochondrial DNA (mtDNA)‐based liquid biomarkers to improve TNBC management. Mitochondrial genome (MG) enrichment and next‐generation sequencing mapped the entire MG in 73 samples (64 tissues and 9 extracellular vesicles [EV] samples) from 32 metastatic TNBCs. We measured mtDNA and cardiolipin (CL) contents, NDUFB8, and SDHB protein expression in tumors and in corresponding circulating EVs. We identified 168 nonsynonymous mtDNA mutations, with 73% (123/186) coding and 27% (45/168) noncoding in nature. Twenty percent of mutations were nucleotide transversions. Respiratory complex I (RCI) was the key target, which harbored 44% (74/168) of the overall mtDNA mutations. A panel of 11 hotspot mtDNA mutations was identified among 19%–38% TNBCs, which were detectable in the serum‐derived EVs with 82% specificity. Overall, 38% of the metastatic tumor‐signature mtDNA mutations were traceable in the EVs. An appreciable number of mtDNA mutations were homoplasmic (18%, 31/168), novel (14%, 23/168), and potentially pathogenic (9%, 15/168). The overall and RCI‐specific mtDNA mutational load was higher in women with African compared to European ancestry accompanied by an exclusive abundance of respiratory complex (RC) protein NDUFB8 (RCI) and SDHB (RCII) therein. Increased mtDNA (*p* < 0.0001) content was recorded in both tumors and EVs along with an abundance of CL (*p* = 0.0001) content in the EVs. Aggressive tumor‐signature mtDNA mutation detection and measurement of mtDNA and CL contents in the EVs bear the potential to formulate noninvasive early detection and recurrence prediction strategies.

## Graphical abstract

The triple‐negative breast cancer patients harbor a racially disparate abundance of mtDNA mutations, traceable in their circulating extracellular vesicles in concert with high mtDNA and cardiolipin (CL) contents. Circulating mtDNA mutation detection and measurement of mtDNA and CL contents bear the potential to formulate noninvasive early diagnostic and recurrence prediction strategies.

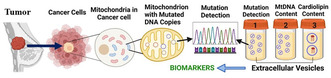

## 1 INTRODUCTION

Triple‐negative breast cancer (TNBC) represents about 15%–30% of all invasive breast cancer cases in the United States.^1^, ^2^, ^3^ Women with African ancestry have a higher prevalence of TNBC with worse clinical outcomes than women with European ancestry.^4^, ^5^, ^6^ Other than the non‐biological factors (lifestyle, socioeconomic status, etc.), several biological factors such as tumor heterogeneity, somatic genomic mutations, and elevated gene expression in breast cancer patients with African ancestry tumors have been attributed to TNBC disparity.^4^, ^5^, ^6^ Cancer cells constantly require a higher amount of energy for recurrent growth and progression. Mitochondria are the key cytoplasmic organelle in the cells to generate energy in the form of ATP.^7^, ^8^ Cancer cells have the remarkable ability to rewire mitochondrial energy generation cascade to their favor and achieve metastatic growth, thereby causing significant mortality. Thus, mitochondria are critical for mitigating metabolic stress and promoting cancer cell survival. In humans, mitochondria are maternally inherited and uniquely process their own DNA (mtDNA, 16.5 kb). The mitochondrial genome (MG) encodes 37 genes, which eventually synthesize 13 proteins to generate ATP through the oxidative phosphorylation system (OXPHOS) in the mitochondria. Due to limited DNA repair capacity, mtDNA is susceptible to damage by environmental carcinogens as well as endogenous reactive oxygen species (ROS). As a result, the mutation rate in mtDNA is much higher compared to nuclear DNA (nDNA). Being a frequently recurring and environmental exposure‐associated disease,^9^ TNBC initiation and progression to lethal disease could be associated with acquired mtDNA mutations translating into altered metabolism and ROS generation. Our current knowledge is limited on the nature and timing of mtDNA alterations along the path of TNBC progression and racial disparity, and how these mtDNA mutations might help drive certain TNBC tumors to become more aggressive. Extracellular vesicles (EVs) are small vesicles released by all cell types.^10^ Recent studies implicated their potential applications in biomarker and therapeutic development for better management of human cancers.^10^, ^11^, ^12^, ^13^, ^14^

In the present study, we have sequenced the entire MG in matched normal and tumor tissues from 32 TNBC patients. In addition, circulating sera‐derived EVs from nine of these patients were also sequenced for mtDNA mutation detection, therefore, a total of 73 samples were analyzed. We have detected 168 nonsynonymous mtDNA mutations in these patients spanning the coding, and noncoding regulatory regions of the MG. Interestingly, mtDNA mutations were detectable in TNBC patients with lymph node (LN) metastasis. Respiratory complex I (RCI) appears to be the mutational hotspot in these patients. The key and hotspot mtDNA mutations were readily detectable in the circulating EVs. We detected an additional 11 mtDNA mutations, exclusively in the EVs of the nine TNBC patients we examined. We also identified a considerable number of homoplasmic, novel, and potentially pathogenic mtDNA mutations in these patients. The overall and RCI‐specific mtDNA mutational burden was higher in the African compared to the European‐TNBC patients, who also exhibited exclusive overexpression of nDNA encoded RCI‐NDUFB8 RCII‐SDHB proteins. In addition, we detected enhanced mtDNA contents in the TNBC tumors and the EVs compared to their normal counterparts. An abundance of cardiolipin (CL) content was also recorded in the circulating EVs of the TNBC patients compared to the cancer‐free control subjects.

## 2 MATERIALS AND METHODS

### 2.1 Biospecimen and ethical statement

We obtained de‐identified archived tumors and matched normal tissue samples from 30 triple‐negative breast cancer patients from the Biospecimen Repository Core Facility (BRCF) at the University of Kansas Medical Center (KUMC). Of the 30 cases, 21 cases were formalin‐fixed paraffin‐embedded (FFPE) tissue samples and nine were fresh‐frozen tissue samples. Paired normal/tumor samples (frozen) from additional two TNBC patients were also collected from the Cooperative Human Tissue Network (CHTN). Matched sera samples from nine patients from the BRCF were also collected. Additional serum samples from nine cancer‐free individuals were also collected. The studies using human specimens were approved by the IRB (protocol #20‐222) at the University of South Alabama. Samples were stored at −80°C or 4°C as appropriate until further use. All the de‐identified subjects have consented to allow for the use of their specimens, and only relevant clinical information including age, race, primary diagnosis, stage, grade, etc., was collected for statistical analyses. All methods were performed following the relevant guidelines and the regulations of the University of South Alabama. The demographic information of all the subjects along with the type of samples utilized from each patient is presented in Table S1.

### 2.2 Genomic DNA isolation and mitochondrial DNA enrichment in tumor tissues

For genomic DNA (gDNA) isolation from the frozen normal/tumor samples (*n* = 22, Table S1), we used QIAamp Fast DNA Tissue Kit and protocol (#51404, Qiagen). For the FFPE tissues (*n* = 42), we used seven 10‐μm curls followed by gDNA isolation using the QIAamp DNA FFPE Tissue Kit and protocol (#56404, Qiagen). DNA isolation was carried out on the automated nucleic acid extraction station QIAcube Connect (Qiagen) and DNA integrity was checked using QIAxcel DNA Screening Kit on the QIAxcel advanced instrument. In the next step, mtDNA was enriched from all the samples (*n* = 64) using the REPLI‐g Mitochondrial DNA Kit and protocol (#151023, Qiagen) as described.^15^

### 2.3 Isolation of EVs from serum samples

Of the 32 TNBC patients, serum samples were available from nine cases as presented in Table S1. We isolated EVs from ~200 μL of sera from these patients. The EVs were also isolated from ~200 μL of sera from cancer‐free individuals. The EVs were isolated utilizing size exclusion chromatography per the manufacturer's specifications (#SSEC100A‐1, System Biosciences).^15^ The EVs were suspended in 500 μL of sterile PBS and kept at −80°C.

### 2.4 Enrichment of mitochondrial DNA in EVs

gDNA was isolated from EVs using a commercially available kit specific for isolating gDNA from EVs following the manufacturer's protocol. (# XCF200A‐1, System Biosciences). From the gDNA pool of each patient, we enriched mtDNA using REPLI‐g Mitochondrial DNA Kit (QIAGEN), which utilizes multiple displacement amplification technologies.^15^, ^16^ The integrity and the quality of the amplified mtDNA were verified by PCR analysis and agarose gel electrophoresis before downstream analysis.

### 2.5 Mitochondrial DNA sequencing, variant calling, and data analysis

We utilized 200 ng of highly enriched mtDNA for subsequent next‐generation sequencing (NGS) analysis from all the samples (*n* = 73). Variant Pro Amplicon Sequencing (Variant Pro™) platform was used for NGS of the entire MG (Table S2). The mtDNA capture and the library preparation were carried out in two PCR steps as described earlier.^15^ Subsequently, the resulting libraries were sequenced on the Illumina NovaSeq PE150 platform. After performing QC of the sequenced data using Trimmomatic‐0.38, the clean data were aligned to reference genome NC_012920.1 (https://www.ncbi.nlm.nih.gov/nuccore/251831106) using Bwa‐0.7.12. The alignment was corrected using GATK‐3.8‐0, the variant calling was conducted using ATK‐3.8‐0, Samtools‐1.9, and Varscan‐v2.4.3, and the annotation of the variants was performed using Annovar‐201,707 and snpEff‐4.3i. For a variant call, all the resulting mtDNA sequence variants were interrogated at different human MG databases as described.^17^ We considered sample coverage with more than 1000‐fold (all mapped read). The obtained data were further examined for correction of missing values and discarding sequence variants called with <100 reads. Minor allele frequencies (MAFs) were determined for all mtDNA‐encoded molecules in various groups. For MAF, we first calculated the percent reads for both the wild‐type (i.e., reference) allele and the mutant alleles for each base pair in the MG using MitoSeek. We then calculated MAF as a number of mutant reads/number of wild‐type reads (i.e., reads where the base pair was the same in normal controls and tumor samples) * 100. All the histopathologically confirmed invasive TNBC samples were compared with their matched normal tissue controls to determine the presence of a somatic mutation. A sequence variant was called a somatic mutation when present only in cancer compared to the reference genome and matched normal tissues following specific criteria, that is comparing the variants with existing mitochondria genome databases to exclude known normal variants used for haplotyping.^17^ The haplotype of each subject was determined using the Haplogrep 2.4.0 program.^18^ Pathogenicity and novelty of the mtDNA sequence variants were determined using the Mitomap database.^17^ For calculating mutational load in African and European‐TNBC patients, the number of overall or region‐specific mtDNA mutations was divided by the number of patients from each group.

### 2.6 Western blot analysis

Protein extraction and western blotting were performed following a method as described earlier.^19^ Briefly, cells were washed once with cold phosphate‐buffered saline (PBS) (Corning) and scraped into RIPA lysis buffer (Thermo Fischer Scientific) containing protease and phosphatase inhibitors. Cell lysates were sonicated and subsequently centrifuged at 13,400 *g* for 15 min at 4°C and supernatants were collected. Protein samples were quantified and subsequently resolved by electrophoresis on SDS–PAGE, transferred onto polyvinylidene difluoride (PVDF) membrane (Sigma Aldrich) and subjected to standard immunodetection procedure using specific antibodies: NDUFB8 (1:1000) (Abcam, #192878), SDHB (1:1000) (Abcam, #175325), and *β*‐actin (1:20000) (Cell Signaling, #12262). Secondary antibodies (Cell Signaling) were used at 1:2000 dilutions. Blots were processed with SuperSignal West Femto Maximum Sensitivity Substrate Kit (Thermo Fischer Scientific, #34096) and the signal was detected using a ChemiDoc Imaging System (Bio‐Rad). Densitometry was done using Fiji (ImageJ) software from the National Institutes of Health.

### 2.7 In silico analysis of NDUFB8 and SDHB expression

We examined the mRNA expression pattern of *NDUFB8 and SDHB* in TNBC and various other breast cancer subtypes using publicly available datasets from The Cancer Genome Atlas (TCGA) using a web‐based application UALCAN, the University of Alabama (http://ualcan.path.uab.edu/).

### 2.8 Assessment of mitochondrial DNA content in the tumors and EVs

We measured mtDNA content using gDNA (20 ng) from matched normal and tumor tissues of the 32 TNBC patients (*n* = 64) by real‐time quantitative PCR. To amplify mtDNA, we used MG‐encoded *ND1* (*MT‐ND1*), *MT‐CYTB*, and nDNA‐encoded *GAPDH*, as described earlier.^15^ The mtDNA/nDNA (*MT‐ND1/GAPDH* and *MT‐CYTB/GAPDH*) ratios from triplicate wells of each subject were utilized to plot relative abundance in different groups. In addition, we also amplified mtDNA in the sera‐EVs from nine TNBC and nine cancer‐free control subjects using the multiple displacement amplification as described above using equal amounts of gDNA template. MtDNA fold enrichment was determined by the Qubit fluorometer quantitation system (ThermoFisher).

### 2.9 Measurement of cardiolipin content in the EVs

We measured CL content (#ab241036, Abcam) in the EVs isolated from the sera of nine TNBC patients and nine cancer‐free subjects as described earlier.^15^ In brief, EVs were suspended in 50 μL of CL assay buffer. In the next step, 50 μL of the EVs sample suspended in CL assay buffer was mixed with 50 μL of CL probe and incubated for 5–10 min at room temperature. Finally, we measured fluorescence intensity at Ex/Em 340/480 per the manufacturer's specification. For data normalization, we determined protein concentration, and the CL content was presented as nmol/mg protein.

### 2.10 Statistical analysis

We employed Student's *t*‐tests χ^2^, one‐way ANOVA or Fisher's exact tests as appropriate for statistical analyses. The *p*‐values were two‐sided with confidence intervals at the level of 95%. Computation for all the analysis was performed using the GraphPad Prism program.

## 3 RESULTS

### 3.1 Mitochondrial DNA mutations are frequent in women with TNBC and can be detected at early stages

**FIGURE 1:**
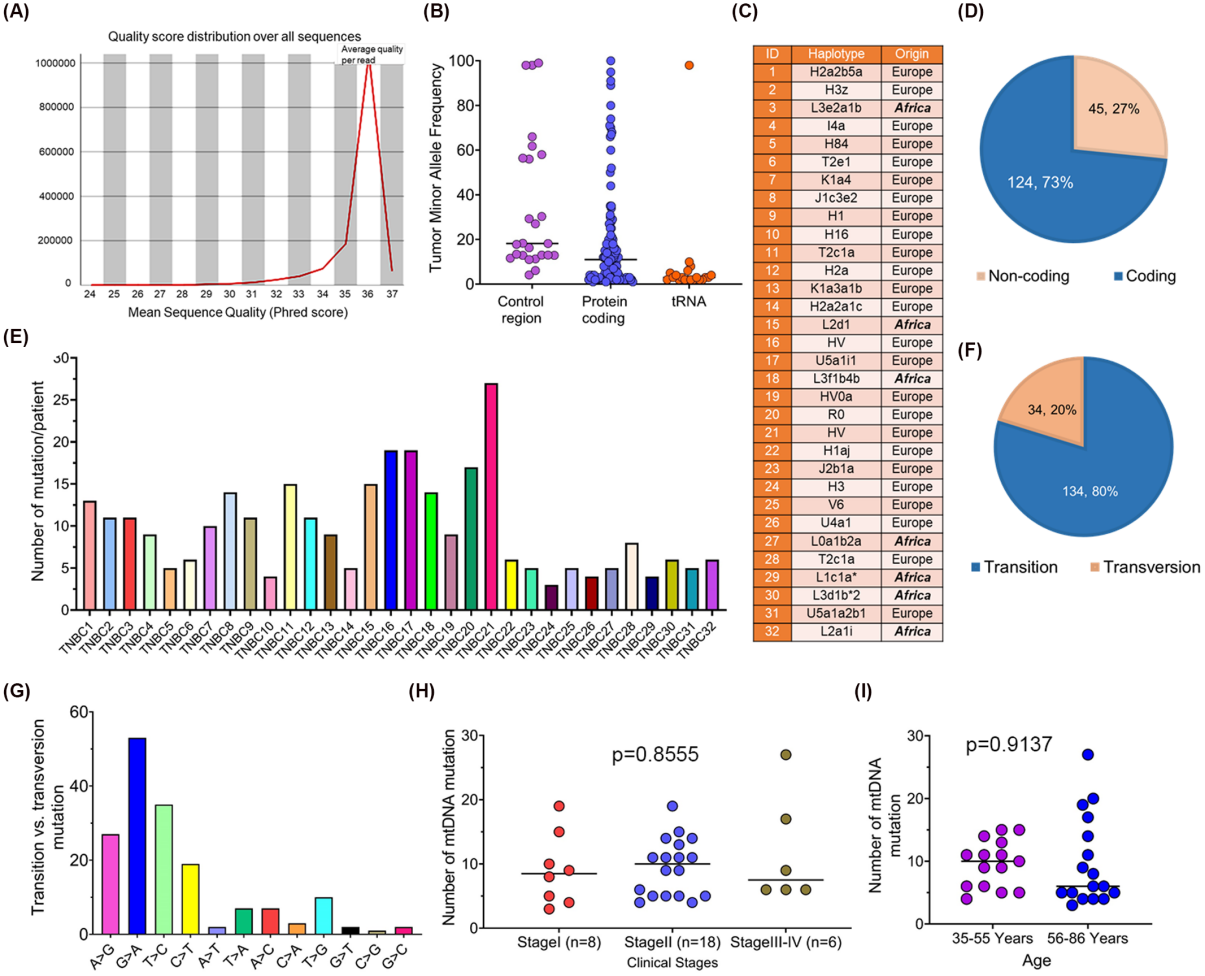
The spectrum of mtDNA sequencing outcomes and clinical association in the TNBC patient. (A) Quality of mtDNA sequencing. (B) Tumor minor allele frequency (MAF) in triple‐negative breast cancer patients in various regions of the mitochondrial genome in the triple‐negative breast cancer patients. (C) mtDNA‐specific haplotype of the TNBC patients. (D) Distribution of the mitochondrial DNA mutations in coding and noncoding regions. (E) The number of mitochondrial DNA mutations in each patient. (F) Transition versus transversion mitochondrial DNA mutations among the triple‐negative breast cancer patients (G) Representation of the type of transition and transversion mutations among the subjects. (H) Association between stage and mitochondrial DNA mutation, (I) The correlation between patients' age and mitochondrial DNA mutations.

We sequenced mtDNA in 73 tissue samples collected from 32 women with a primary diagnosis of TNBC. The quality of the sequence reads and the tumor MAFs of all the samples have been presented in Figure 1A,B. We also determined the ancestry of each patient based on the haplotype‐specific mtDNA markers (Figure 1C). We have recorded 168 nonsynonymous mtDNA mutations in all the patients across various coding and noncoding regions of the mtDNA. Of these mutations, 73% (124/168) were from the coding regions and 27% (45/168) were from the noncoding regions of the mtDNA (Figure 1D). At least, three nonsynonymous mtDNA mutations were detected in each patient (Figure 1E). Eighty percent (134/168) of the mutations were nucleotide transitions, whereas 20% (34/168) were nucleotide transversions (Figure 1F). The G > A transition mutation was the highest among all the patients followed by T > C, A > G, C > T, and other transversion mutations (Figure 1G). The mtDNA mutations were detectable at the early stage and in young age (Figure 1H,I), although it did not reach a statistically significant level in terms of a correlation (*p* = 0.62 and 0.91, respectively).

### 3.2 RCI is the key target of mitochondrial DNA mutations in TNBC progression

**FIGURE 2:**
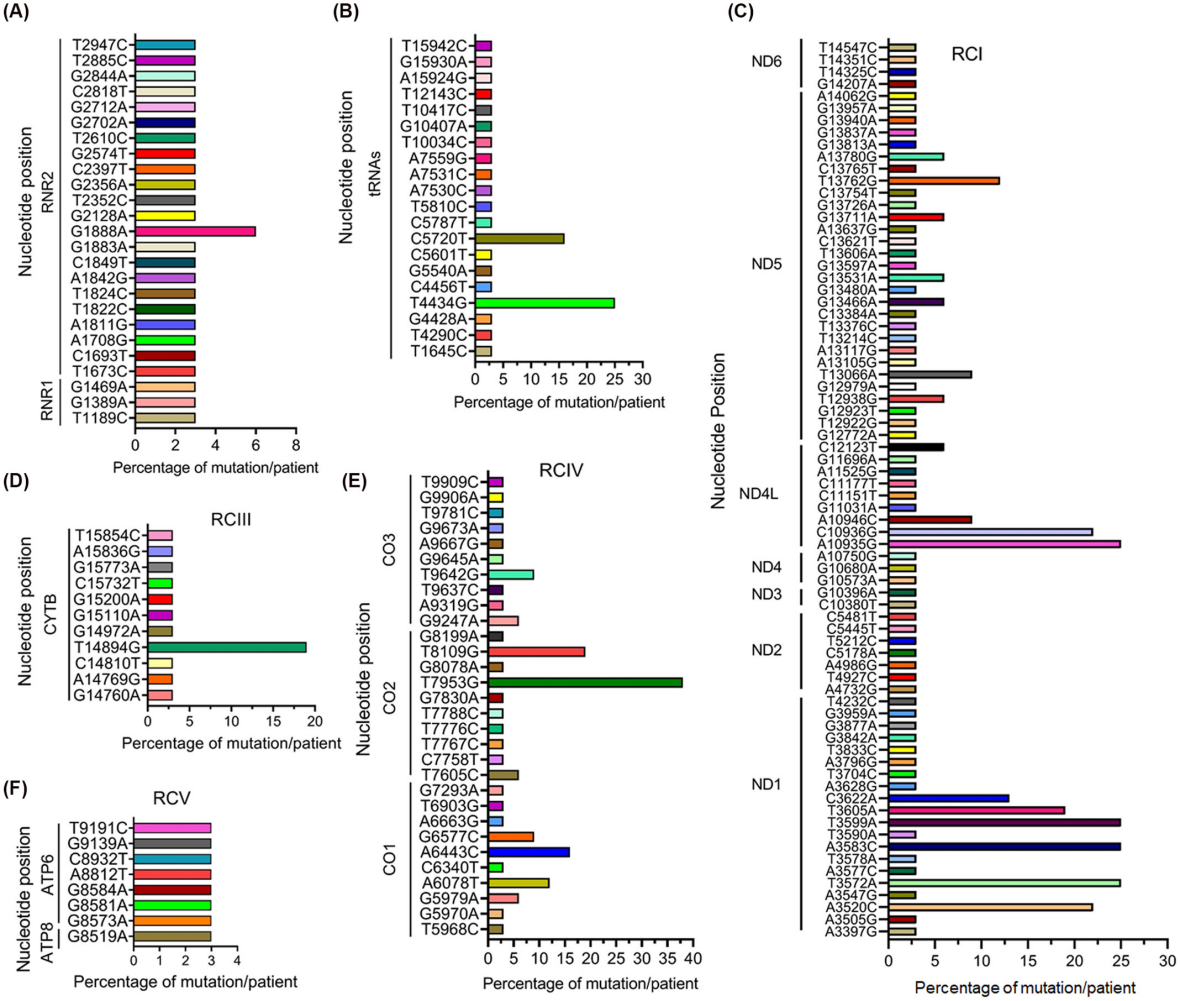
The pattern of mitochondrial DNA mutations in the noncoding and coding regions of the mitochondrial genome. (A) Distribution of mitochondrial DNA mutations in the control regions as indicated. (B) The pattern of mitochondrial DNA mutations in the tRNA region as indicated. The percentage of each mutation was calculated based on the number of observed mutations per patient at each specific nucleotide position. RNR1: 12S RRNA; RNR2: 16S RRNA. (C) Distribution of mitochondrial DNA mutations in the respiratory complex I (RCI) as indicated. (D) Mitochondrial DNA mutations in the respiratory complex III (RCIII, *MT‐CYTB*). (E) The pattern of mitochondrial DNA mutations in the respiratory complex IV (RCIV). (F) Location of mitochondrial DNA mutations in the respiratory complex V (RCV) as indicated. The percentage of each mutation was calculated based on the number of observed mutations per patient at each specific nucleotide position. ND: NADH dehydrogenase; NADH:Ubiquinone oxidoreductase; CYTB: Cytochrome *B*; CO: Cytochrome *c* oxidase subunit; ATP6: ATP synthase F0 subunit 6; ATP8: ATP synthase F0 subunit 8.

Next, we examined the distribution pattern of the mtDNA mutation across the entire MG of all the TNBC patients. We observed ~15% (25/168) mtDNA mutations in the noncoding regulatory region involving *RNR1* and *RNR2* and ~ 12% (20/168) mutations spanning the *tRNA* genes. The frequency of the *RNR1* and *RNR2*‐mtDNA mutations in the TNBC patients was 3% except for one mutation (G1888A), which was detected in 6% of patients (Figure 2A). In comparison, the frequency of the tRNA‐specific mtDNA mutations in the TNBC patients was 3% except for a couple of mutations, which were detected in 16% (C5720T) and 20% (T4434G) patients, respectively (Figure 2B). The rest of the mutations were distributed among the mtDNA encoded RCI (*MT‐ND1*, *ND2, ND3, ND4, ND4L, ND5*, and *ND6)*, RCIII (*MT‐CYTB*), RCIV (*MT‐CO1, CO2* and *CO3*), and RCV (*MT‐ATP6* and *ATP8*) subunits. Among these coding mtDNA mutations, 44% (74/168) were from the RCI (Figure 2C). Among the 74 RCI mutations, 20 were from *MT‐ND1*, seven were from *MT‐ND2*, two were from *MT‐ND3*, three were from *MT‐ND4*, nine were from *MT‐ND4L*, 29 were from *MT‐ND5*, and four were from *MT‐ND6*. The frequency of detection of the RCI‐mtDNA mutations in the TNBC patients ranged between 3% and 25%. Approximately 7% of mtDNA mutations (11/168) were detected in the RCIII region represented by a single gene *MT‐CYTB* (Figure 2D). The frequency of the RCIII‐mtDNA mutation in the TNBC patients was 3% except for one mutation (T14894G), which was detected in 20% of the patients. We detected 18% of the mtDNA mutation (30/168) in the RCIV region. Among these 30 mutations, 10 each was identified in *MT‐CO1*, *MT‐CO2*, and *MT‐CO3* region (Figure 2E). The frequency of the RCIV‐mtDNA mutation in the TNBC patients ranged between 3% and 20% except for one mutation (T7953G), which was detected in 38% of the patients. In addition to the above RCs, a 5% mutation (8/168) was observed in RCV (Figure 2F). Of the eight RCV mutations, seven were from the *MT‐ATP6* and one was from the *MT‐ATP8* region. The frequency of the RCV‐mtDNA mutation in the TNBC patients was 3%.

### 3.3 The TNBC patients harbor hotspot, novel and potentially pathogenic mitochondrial DNA mutations, traceable in the circulating EVs

**FIGURE 3:**
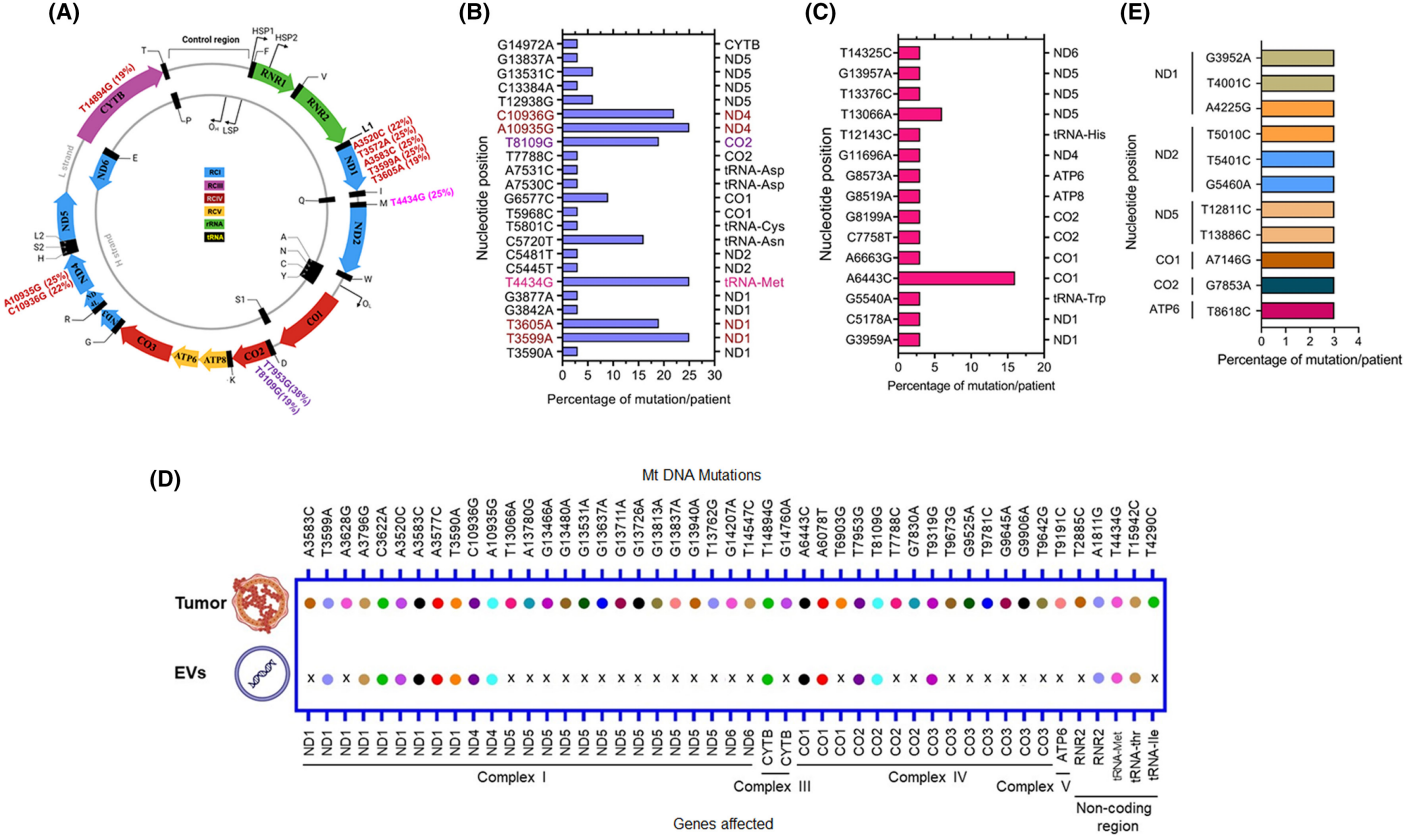
Hotspot, pathogenic, and novel mtDNA mutations in the tumors and their detection in the circulation. (A) Panel of hotspot mitochondrial DNA mutations frequently detected among the TNBC patients. The mutations from various respiratory complexes are represented in different colors along with their frequency of occurrences. The image was adapted from Biorender.com and modified as necessary. The separate 22 letters represent 22 mitochondrial tRNAs. HSP: H strand promoter; LSP: Light strand promoter; ND: NADH dehydrogenase; CYTB: Cytochrome B; CO: Cytochrome *c* oxidase; ATP6: ATP synthase F0 subunit 6; ATP8: ATP synthase F0 subunit 8; MT: Mitochondrial; RC: Respiratory complex. (B) Novel mitochondrial DNA mutations, observed in the TNBC patients in various coding and noncoding regions of the mitochondrial genome. The matched color indicates the novelty of the hotspot mtDNA mutations. (C) Potentially pathogenic DNA mutations detected in the coding and noncoding regions of the mitochondrial genome as indicated utilizing the Mitomap database. ND: NADH dehydrogenase; CYTB: Cytochrome *B*; CO: Cytochrome *c* oxidase; ATP6: ATP synthase F0 subunit 6; ATP8: ATP synthase F0 subunit 8; Asp: Aspartic acid; Cys: Cysteine; Met: Methionine; His: Histidine; Asn: Asparagine; Trp: Tryptophan. (D) Detection of mitochondrial DNA mutations in extracellular vesicles (EV) of nine TNBC patients. Each mutation has been represented in a different color and the corresponding nucleotide alterations and affected genes were indicated. X indicates when a mutation is not detected in a specific sample. The representative nucleotide sequence of tumor‐matched EVs was compared with the matched normal and the reference genome sequence (NC_012920.1). The nature of mtDNA mutations observed in the coding and the noncoding regions of the mtDNA has been depicted. RNR2: 16S RRNA; ND: NADH dehydrogenase; CYTB: Cytochrome B; CO: Cytochrome *c* oxidase; Met; Methionine; Thr: Threonine. Tumor, EV, and serum images were adapted from Biorender.com. (E) Exclusively detected mtDNA mutations in the EVs of the TNBC patients when compared to the reference sequence, corresponding normal, and tumor samples. RC: Respiratory complex; ND: NADH dehydrogenase; CO: Cytochrome *c* oxidase; ATP6: ATP synthase F0 subunit 6.

As mentioned above, 168 different types of somatic mtDNA mutations were detected in 32 unique TNBC cases. In the next step, we determined the frequency of occurrence of these mutations across different patients. We identified a panel of 11 hotspot mtDNA mutations, which were detected in 19%–38% of the patients (Figure 3A). Among these 11 mtDNA mutations, 10 were present in the protein‐coding region. Of these coding mtDNA mutations, 70% (7/10) were from RCI, 20% (2/10) were from RCIV, and 10% (1/10) were from RCIII. We also identified a panel of novel (Figure 3B) and potentially pathogenic (Figure 3C) mtDNA mutations in these patients. Specifically, 23 novel mtDNA mutations were found, among which, 56% (13/23) were from the RCI, 17% (4/23) were from the RCIV, 4% (1/23) were from RCIII, and 28% (5/18) were from the tRNA region. Six out of twenty‐three novel mutations (26%) belong to the hotspot panel (Figure 3A). There were 15 potentially pathogenic mtDNA mutations detected, of which 47% (7/15) were from RCI, 27% (4/15) were from the RCIV, and14% (2/15) were from RCV, and tRNA regions of the mtDNA.

To identify whether tumor‐signature mtDNA mutations are traceable in the circulation, we enriched and sequenced mtDNA from sera‐derived EVs from nine of the TNBC cases (Table S1). These nine patients (#8, #9, #14, #15, #23, #25, #26, #27, and #31) exhibited 45 mtDNA mutations encompassing the coding and noncoding regions of the mtDNA in their primary tumors (Figure 3D). All nine TNBC patients were detected with at least one mtDNA mutation in the EV that was present in their primary tumors. Of the 45 mtDNA mutations detected in the primary tumors, 38% (17/45) were detectable in the EVs enriched from the circulating sera of these patients (Figure 3D). Notably, 82% (9/11) of the tumor‐signature hotspot‐mtDNA mutations (Figure 3A) were readily detectable in the circulating EVs of the TNBC patients. In addition, we also detected a set of 11 mtDNA mutations present exclusively in the circulating EVs of the TNBC patients (Figure 3E). Of these 11 mutations, 73% (8/11) was from RCI, 18% (2/11) was from the RCIV, and 9% (1/11) was from RCV region of the mtDNA. Of these mutations, T4001C (*MT‐ND1*) appeared to be novel.

### 3.4 Nuclear‐encoded RCI and RCII subunits are overexpressed in TNBC patients with African ancestry

**FIGURE 4:**
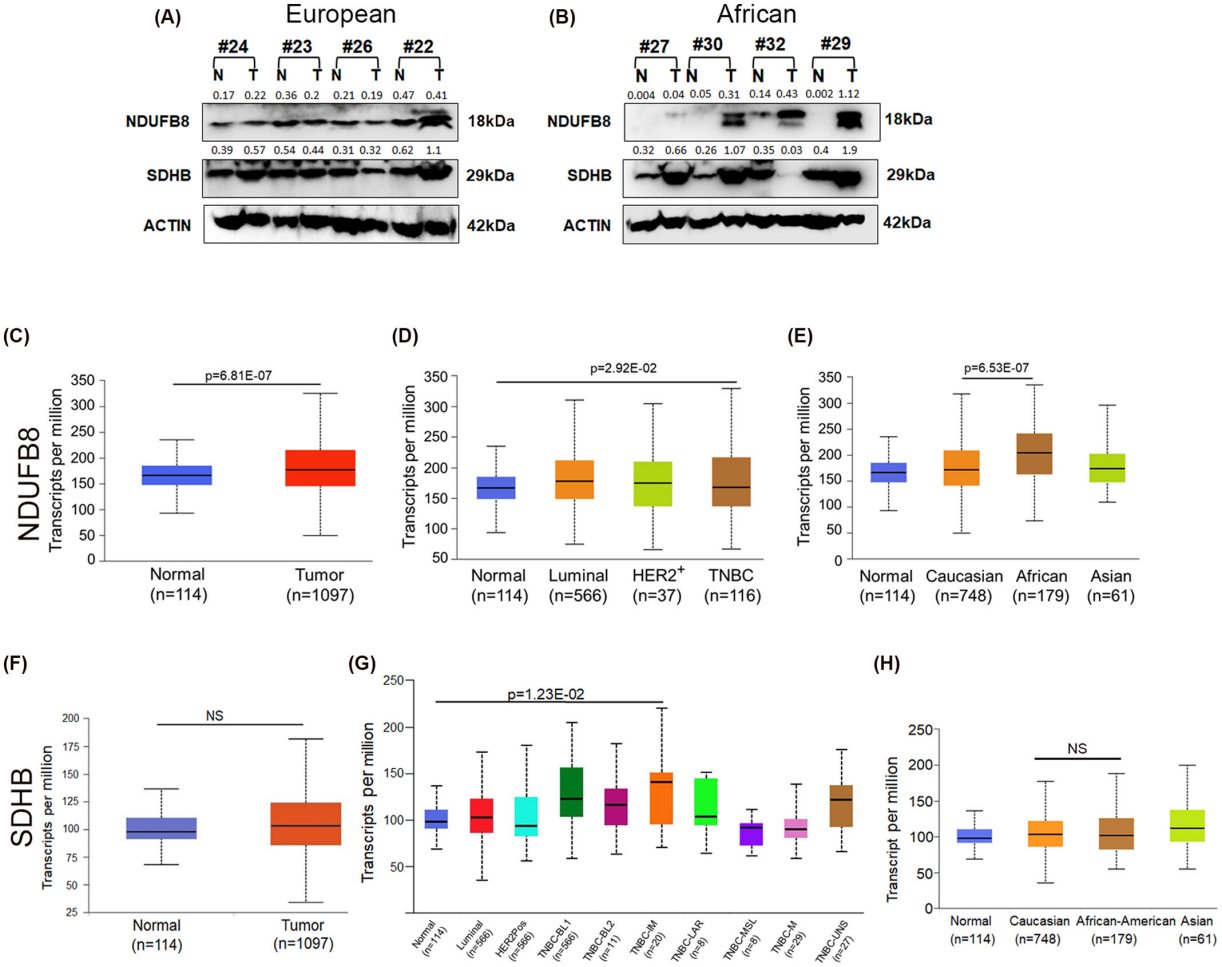
Nuclear DNA‐encoded respiratory complex subunit expression in TNBC patients. (A) The expression pattern of NDUFB8 and SDHB proteins in TNBC patients with European ancestry (#22, #23, #24, and #26). (B) NDUFB8 and SDHB protein expression in TNBC patients with African ancestry (#27, #29, #30, and #32). Actin was used as the loading control. (C) The mRNA expression pattern of *NDUFB8* in overall breast cancer patients. (D) The mRNA expression pattern of *NDUFB8* in different breast cancer subtypes including TNBC. (E) *NDUFB8* mRNA expression pattern in breast cancer patients with divergent ancestral origins. (F) The mRNA expression pattern of *SDHB* in overall breast cancer patients. (G) The mRNA expression pattern of *SDHB* in different breast cancer subtypes including TNBC. (H) *SDHB* mRNA expression pattern in breast cancer patients with various ancestral backgrounds. The mRNA expression data were adapted from the UALCAN database, the University of Alabama.

In this study, the majority of the mtDNA mutations were detected in the RCI region. We measured the protein expression pattern of a key RCI subunit, NDUFB8 (NADH:Ubiquinone Oxidoreductase Subunit B8), which is nuclear‐encoded and involved in mitochondrial RCI assembly. In parallel, we also measured the expression pattern of SDHB (Succinate Dehydrogenase Complex Iron Sulfur Subunit B), a key subunit of RCII. Of the eight TNBC patients that we have examined, markedly higher expression of NDUFB8 was noted (3‐ to 56‐folds) in 50% (4/8) of the patients (Figure 4A,B). Notably, these four patients (#27, #29, #30, and #32) with high *NDUFB8* expression had African ancestry and were from Stages I‐III (Table S1 and Figure 1C) similar to the stages of the patients with European ancestry (#22, #23, #24, and #26, Stages I‐III) (Table S1 and Figure 1C). Interestingly, we detected a potential transcript variant of NDUFB8 exclusively in TNBC patients with African ancestry. We also observed a comparatively higher expression (threefold) of NDUFB8 in a TNBC cell line MDA‐MB‐468 developed from a woman with African ancestry compared to the MDA‐MB‐231 cell line, generated from a TNBC patient with European ancestry (Figure S2). NDUFB8 expression was barely detectable in the non‐tumorigenic MCF‐10A breast epithelial cell line generated from a woman with European ancestry. Notably, the additional NDUFB8 variant we observed in the African‐TNBC patients (Figure 4B), was appreciably detectable in the African‐MDA‐MB‐468 cell line and faintly in the MDA‐MB‐231 cell line (Figure S2). At the same time, we observed higher expression (two‐ to fivefold) of SDHB subunit in 50% (4/8) of the patients. One patient had shown a notable decrease in SDHB expression (#32, 12‐fold). Three out of the four patients with high *NDUFB8* expression also exhibited a higher level of SDHB expression. Notably, the mean age of the four African‐TNBC patients was 53.25 years, whereas the mean age of the four European patients was 63.5 years. We also examined the mRNA expression pattern of *NDUFB8* and *SDHB* using publicly available TCGA datasets from TCGA. We observed significant overexpression of *NDUFB8* mRNA (*p* = 6.81E‐07) in breast cancer patients (*n* = 1097) compared to their normal counterparts (*n* = 114) (Figure 4C). A significantly higher expression of *NDUFB8* (*p* = 2.92E‐02) was also noted in TNBC patients (*n* = 116) compared to the same normal control samples (Figure 4D). Notably, *NDUFB8* mRNA expression was also found to be higher (*p* = 6.53E‐07) in the African (*n* = 179) compared to the Caucasian (*n* = 748) breast cancer patients (Figure 4E). On the other hand, SDHB mRNA expression in overall breast cancer patients (*n* = 1097) was higher compared to the normal (*n* = 114), however, it did not reach a significant level (Figure 4F). However, significantly higher expression of SDHB transcripts was evident in the immunomodulatory subtypes of TNBC compared to the normal controls (Figure 4G, *p* = 1.23E‐02). In comparison, a similar pattern of *SDHB* mRNA expression was evident in the breast cancer patients with African (*n* = 179) and European (*n* = 748) ancestry (Figure 4H).

### 3.5 TNBC patients with African ancestry have higher mtDNA mutation load

**FIGURE 5:**
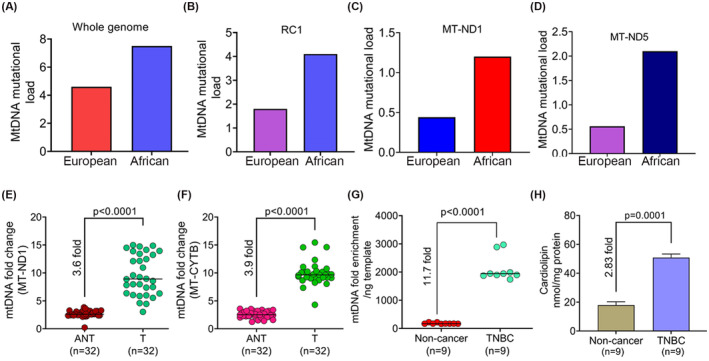
The racial distribution of mitochondrial DNA mutations along with mitochondrial DNA and cardiolipin (CL) content in extracellular vesicles (EVs). (A) Overall load of mitochondrial DNA mutations in TNBC patients with European and African Ancestry. (B) Load of respiratory complex I targeted mitochondrial DNA mutations in African and European‐TNBC patients. (C) Load of mitochondrial DNA mutations in the *MT‐ND1* gene among the TNBC patients with African and European ancestry. (D) Mitochondrial DNA mutations in the *MT‐ND5* region among the TNBC patients of African or European ancestry. (E,F) Relative abundance of mtDNA content in the TNBC tumors (T) compared to their adjacent normal tissue (ANT). GAPDH was used as a nuclear DNA (nDNA) control. A ratio of mtDNA/nDNA from triplicate wells represents fold change in each case. (G) Mitochondrial DNA fold enrichment in the EVs of the TNBC patients compared to the cancer‐free controls. (H) CL contents in the EVs of the TNBC patients and the cancer‐free controls. The CL contents were measured in an equal amount of sera‐derived EVs of the TNBC and the noncancer subjects.

Among the 32 TNBC subjects, seven were of African ancestry, whereas 25 patients had European ancestry. Considering the overall mtDNA mutations, the mutational load was higher among the African‐TNBC compared to the European‐TNBC patients (7.5 vs. 4.6 mutations) (Figure 5A). We have observed the most frequently occurring mtDNA mutations in the RCI region. The mutation load in RCI was also found to be higher in the African compared to the European Group (4.1 vs. 1.8 mutations) (Figure 5B). In the RCI, most of the mutations were recorded in *MT‐ND5* and *MT‐ND1* genes. Mutation load in *MT‐ND1* and *MT‐ND5* was higher in the African compared to the European‐TNBC patients (1.2 vs. 0.44‐MT‐ND1; 2.1 vs. 0.56‐MT‐ND5) (Figure 5C,D). Notably, homoplasmic mtDNA mutational load was also recorded as slightly higher among women with African ancestry (1.4 vs. 0.84 mutations). However, the majority of the RCI‐hotspot mutations (A3520C, T3572A, T3599A, and T3605A in *MT‐ND1* and A10935G in *MT‐ND4*) were shared between the TNBC patients with African and European ancestry. Similar was the case for the T4434G mutation in the *tRNA‐Met* gene.

### 3.6 Higher mtDNA content in tumors and EVs along with abundant CL content in the circulation of the TNBC patients

Changes in mtDNA and CL contents could be associated with altered mitochondrial function. We first measured mtDNA content in the matched normal and tumor tissues of all 32 TNBC patients. The mtDNA content was significantly higher (3.6–3.9 fold, *p* < 0.0001) in the tumors of the TNBC subjects compared to their adjacent normal tissues (Figure 5E,F). Similarly, we recorded a significant enrichment of mtDNA in the sera‐derived EVS of the 9/32 TNBC patients (Table S1) compared to the cancer‐free controls (11.7 fold, *p* < 0.0001) (Figure 5G). We also observed a considerably higher CL content (2.83 fold, *p* = 0.0001) in the EVs of the nine TNBC patients compared to the cancer‐free subjects (Figure 5H and Figure S1).

### 3.7 The high‐grade TNBC patients harbor homoplasmic mitochondrial DNA mutations

**FIGURE 6:**
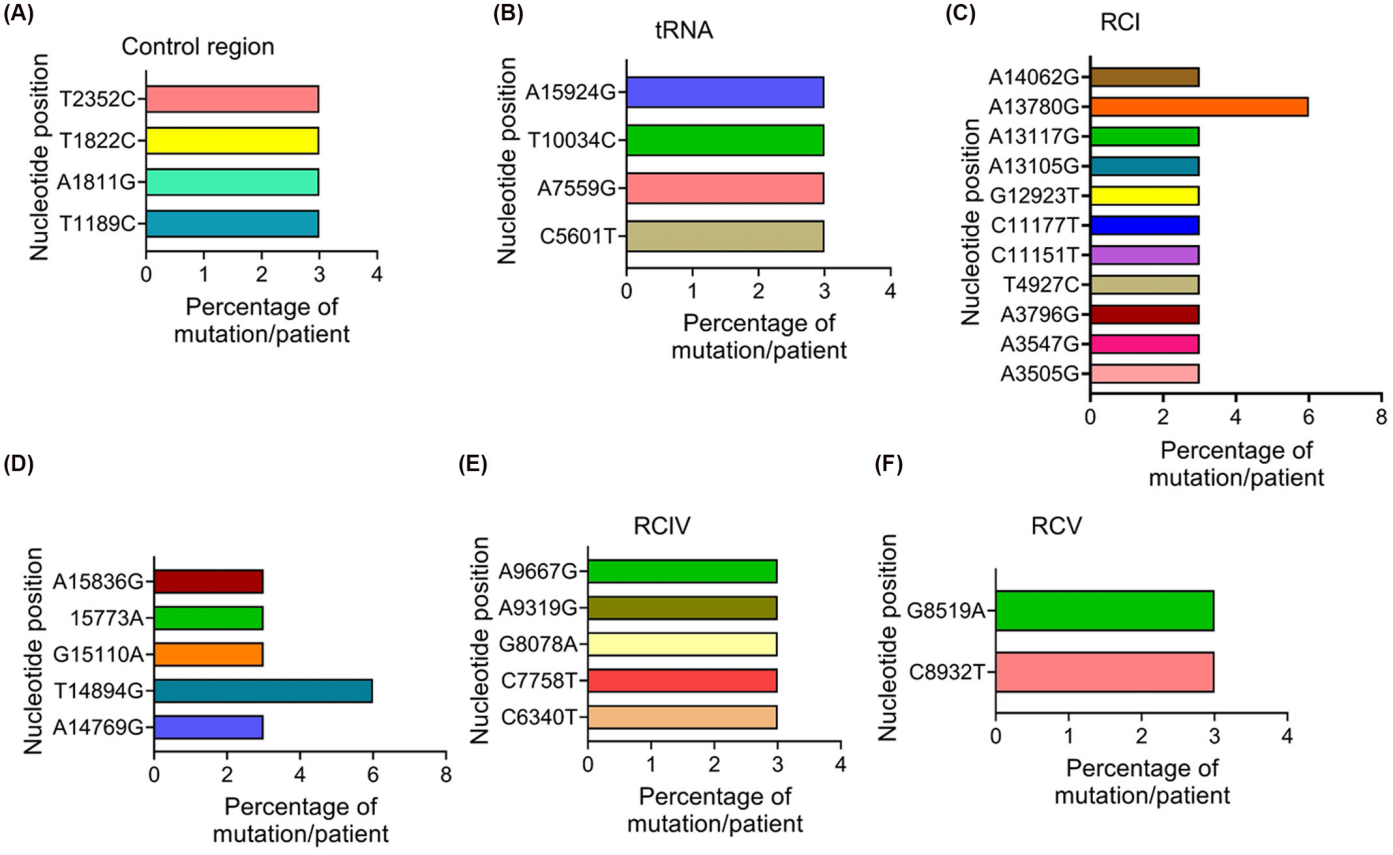
Analysis of homoplasmic mtDNA mutations in TNBC patients. Distribution of percent homoplasmic mitochondrial DNA mutation in the (A) control region (B) tRNA (C) RCI (D) RCIII, (E) RCIV, and (F) RCV across the mitochondrial genome in TNBC patients.

Homoplasmic mutations could be pathogenic and bear functional significance. Among the 168 mtDNA mutations that we have identified in this study, 18% (31/168) were homoplasmic in nature. Among the noncoding mutations, 13% each (4/31) were detected in both the control (Figure 6A) and tRNA (Figure 6B) regions of the mtDNA. Among the coding homoplasmic mtDNA mutations, 34% (11/31) were recorded in the RCI (Figure 6C), 16% each (5/31) were in RCIII (Figure 6D) and RCIV (Figure 6E) regions and 6.5% (2/31) were in the RCV (Figure 6F) regions. Four homoplasmic mtDNA mutations (*G8519A‐MT‐ATP8, C6340T‐CO1, C7758T‐MT‐CO2, and G8078A‐MT‐CO2*) appeared to be potentially pathogenic in nature (Figure 3C). The overall frequency of the homoplasmic mtDNA mutation in the TNBC patients was 3% except for two RCI and RCIII mutations, which were detected in 6% of the patients.

## 4 DISCUSSION

Despite recent advancements in the “multi‐omics” understanding of tumorigenesis, early detection of the lethal lesions, their recurrence prediction, and therapeutic management of TNBC patients are significant clinical challenges. The majority of TNBC patients are diagnosed at advanced stages, predominantly in young women with African ancestry.^1^, ^2^, ^3^, ^4^, ^5^, ^6^ The late‐stage diagnosis, when tumors achieve metastasis as evident in our cohorts, significantly limits therapeutic interventions, thereby resulting in the worst survival. The mitochondrial OXPHOS system plays a significant role in maintaining the energy balance of aggressively growing tumors.^20^ The MG encoded machinery, constitutes the critical components of this OXPHOS pathway to generate 90% of the cellular ATP.^7^ In concert with the nDNA‐encoded multiple RC proteins, the MG thus plays a pivotal role in driving the OXPHOS pathway and fueling tumor growth and progression. Tumor‐promoting roles of several nDNA encoded genes' mutations such as *EGFR, KRAS, P53*, etc. are well established in human tumorigenesis.^21^, ^22^, ^23^, ^24^, ^25^, ^26^, ^27^, ^28^ As a reason, they are very useful as prognostic biomarkers and for therapeutic management. In agreement, several studies have identified mtDNA mutations in various human cancers including breast carcinomas.^15^, ^16^, ^18^, ^29^, ^30^, ^31^, ^32^, ^33^, ^34^, ^35^, ^36^ Moreover, a few studies have also established the functional significance of the human patients' derived bonafide mtDNA mutations in promoting tumorigenesis.^29^, ^31^, ^32^, ^37^, ^38^, ^39^ In this light, MG appears to have clinical significance and bears potential for biomarker and therapeutic development. However, the pattern of mtDNA mutations in TNBC patients with various ancestral backgrounds and the sensitivity and specificity of mutation detection in circulation remains largely unknown.

TNBCs are diagnosed predominantly at times when tumors already metastasized to the LN(s) and/or various organ sites. Even, the detection of a Stage‐I TNBC tumor with a minimum single LN metastasis is challenging due to the lack of appropriate interventions. Moreover, a Stage‐I tumor does not rule out the possibility of micro‐metastases in multiple LNs and distant organs, which remains undetectable by mammography and currently available methods at the time of first diagnosis. In addition, there are no reliable prognostic biomarkers for accurate monitoring of TNBCs. In this light, frequent detection of potentially pathogenic and hotspot mtDNA mutations in the tumors and the circulating EVs of the TNBC patients with the early onset of metastasis may serve as useful biomarkers for early detection of the lethal lesions, their monitoring, and measuring therapeutic response. At the same time, the detection of the frequently occurring mtDNA mutations in early metastatic TNBCs suggests their potential role in the recurrent and metastatic progression of the disease. Since TNBCs do not progress through the preneoplastic ductal carcinoma in situ (DCIS) in most of the cases and remain undetected without metastasis, we could not analyze those samples for mtDNA mutation detection. However, if the hotspot mtDNA mutations that we cataloged are associated with metastatic dissemination at the earliest time point, they can guide in predicting disease outcomes in advance, leading to improved therapeutic management.

In the OXPHOS system, RCI serves as the entry gate for the electron transport system to begin.^7^ Alterations in RCI may lead to higher ROS production through electron leaking and inhibition of intrinsic apoptosis. In our study, RCI had the highest mutational burden compared to the other coding and noncoding regions of the mtDNA and was the mutational hotspot among TNBC patients. This finding strongly suggests the potential role of RCI‐mtDNA mutations in TNBC pathogenesis. Notably, mutations in RCI were found to be associated with the progression and therapeutic resistance of various malignancies.^40^ In an earlier study, the G3842A mutation in *MT‐ND1* that we detected in the metastatic‐TNBC patients, was found to be associated with hepatocellular carcinoma progression.^41^ The tumors harboring beneficial RC‐mtDNA mutations may also augment the production of nDNA‐encoded relevant RC‐subunits through mitochondria‐nuclear retrograde signaling as a compensatory mechanism to keep up the pace of the OXPHOS system to fulfill high‐energy demand and promote progression. Possibly as a reason, we observed elevated protein expression of nDNA‐encoded RCI and RCII subunits in the TNBC patients. Notably, augmented protein expression of the RCI and RCII (all but one) subunits exclusively in the African‐TNBC patients bearing higher overall, and RCI‐specific mtDNA mutation load indicates a prevalence of enhanced mitochondrial function to promote disparate TNBC aggressiveness. In addition, detection of the potentially new variant of *NDUFB8*, exclusively in the primary tumors and established cell model derived from TNBC patients with African ancestry may have functional implications in racially disparate disease outcomes. Further comprehensive analysis of the variants is thus warranted. Evident loss of RCII‐SDHB expression in one patient reflects heterogeneity between tumor cell clones, which necessitates the development of personalized treatment. Although the noncoding genes do not make proteins, however, mutations in these genes can influence the expression of the coding genes and mitochondria‐nuclear retrograde signaling.^40^

Recent studies suggest that transversion mutations have more potential to hamper transcription factors' binding, thereby causing profound changes in genes' expression.^42^ In this context, the detection of an appreciable frequency of transversion mutations in the mtDNA may have similar implications in promoting TNBC pathogenesis. Although, we identified a panel of potentially pathogenic mtDNA mutations, the likely pathogenicity of the novel mtDNA mutations that we discovered remains to be determined. In various human cancers, e.g., lung adenocarcinomas; *EGFR* gene mutation is present in 10%–20% of the patients with African and European ancestry and thereby serves as an excellent biomarker and therapeutic target.^21^, ^43^, ^44^ The panel of 11 mtDNA mutations, detected in 19%–38% of the TNBC patients, could also be useful in biomarker development and may bear potential in therapeutic targeting. A recent study in breast cancer noted a shift in heteroplasmic mtDNA mutation toward homoplasmy in tumor samples,^34^ which may have functional importance in driving tumorigenicity. Detection of an appreciable number of homoplasmic mtDNA mutations, predominantly in the coding regions of invasive tumors of the TNBC patients further supports this notion.

Early detection, accurate monitoring, and/or surveillance of cancer patients using a noninvasive or minimally invasive approach without requiring the tumor tissues at different time points are ideal for suitable disease management in clinical settings. In this context, circulating EV harboring metastatic tumor‐associated hotspot mtDNA mutations with high specificity could be excellent resources for minimally invasive liquid biomarker development. In a recent study, we detected several tumor‐associated coding and noncoding mtDNA mutations in sera‐derived EVs from pancreatic cancer patients.^15^ Some of these mutations including T3572A, A3577C, A3583C (*MT‐ND1*), A10935G (*MT‐ND4)*, T12922G, T13762G, A13780G (*MT‐ND5*), and A1811G (*RNR2*) were readily detectable in the tumors and matched EV samples of the metastatic‐TNBC patients. Thus, routine analysis of mtDNA mutation detection in EVs from cancer patients of various anatomic origins could be useful for accurate monitoring/surveillance and measuring therapeutic responses. Another study demonstrated the transfer of *wild‐type* MG through EVs from cancer‐associated fibroblasts to the hormone treatment‐resistant, and dormant breast cancer cells, thereby facilitating their progression.^45^ Thus, the EVs carrying mutated mtDNA could potentially promote tumorigenesis by delivering necessary cargo to the neighboring tumor niche. In this light, accurate detection of the tumor‐derived mtDNA mutations in the circulating EVs will not only be useful in minimally invasive biomarker development but also in understanding their role in TNBC tumorigenesis. We have detected a panel of mtDNA mutations exclusively in the circulating EVs of nine TNBC patients, but not in their primary tumors that we examined. Seven of these TNBC patients were in Stage II with metastasis in multiple LNs and two were from Stage I with metastasis in at least one LN. In this light, it is likely that the EVs packaged with an additional set of mutations are released into the circulation from the heterogeneous tumor clones that already metastasize to other LNs after acquiring additional mutations. Notably, all but one of these mutations have been reported in various human cancers and mitochondrial disorders,^46^, ^47^, ^48^, ^49^, ^50^, ^51^, ^52^, ^53^, ^54^, ^55^ which implicate their significance in human tumorigenesis including TNBC, and potentiate their futuristic use in cancer diagnostics.

An increase in mtDNA content was shown to promote colorectal cancer progression by augmenting OXPHOS function.^56^ An association between enhanced mtDNA content and increased risk of breast cancer has also been demonstrated.^57^ Similarly, a comprehensive analysis demonstrated mtDNA abundance in cancer tissues accompanied by increased expression of OXPHOS‐associated genes.^58^ This study also reported an association between the simultaneous increase in mtDNA content and mutations in endometrial carcinomas. Thus, abundant copies of mutated mtDNA are likely to aid in metastatic TNBC progression through augmented metabolic activities. However, functional characterization would be necessary to confirm the pathogenicity of specific mtDNA mutations. On the contrary, decreased copies of mtDNA have also been detected in malignant tumors of various anatomic origins implicating a differential role of mtDNA copy number variation in human tumorigenesis.^58^ CLs have been demonstrated as biomarkers in mitochondria‐enriched thyroid tumors.^59^ In addition to mtDNA, high CL content in the EVs of the metastatic TNBCs further supports their mitochondrial origin as observed in pancreatic cancer patients and rationalizes their use in liquid biomarker development.^15^

In summary, this is the first study cataloging the outcome of entire MG sequencing in tumor tissues and circulating EVs from metastatic‐TNBC patients with divergent ancestral backgrounds. Comprehensive profiling of the panel of hotspot mtDNA mutations in the tumors and their highly sensitive and accurate detection in the circulation of a larger TNBC cohort are warranted. Alongside periodic mammographic evaluations, the formulation of a circulating mtDNA mutation detection system in concert with the measurement of mtDNA and CL contents from a small amount of samples is feasible. This cost‐effective simple approach could open novel avenues for the early detection of potentially lethal lesions, their recurrence prediction, and the prevention of metastatic progression particularly in racially disparate populations.

## AUTHOR CONTRIBUTIONS

**K.S. Vikramdeo:** Data curation, investigation, methodology manuscript editing. **S. Anand**: Data curation, methodology, and manuscript editing. **S.K. Sudan:** Data curation, methodology, and manuscript editing. **P. Pramanik**: Data curation, methodology, reviewing, and manuscript editing. **S. Singh**: manuscript editing, reviewing, and investigation. **A. Singh**: Manuscript editing, reviewing, and investigation. **A.K. Godwin**: Manuscript editing, reviewing, resources, and investigation. **S. Dasgupta**: Conceptualization, data curation, formal analysis, resources, supervision, funding acquisition, investigation, methodology, writing original draft, reviewing and editing, and project management.

## FUNDING INFORMATION

This research was supported by funding from the Breast Cancer Research Foundation of Alabama (BCRFA), Mitchell Cancer Institute and the University of South Alabama to SD. The BRCF is supported in part by the KU Cancer Center's Support Grant (P30 CA168524). The authors would also like to thank all the patients for providing their precious samples.

## CONFLICT OF INTEREST STATEMENT

The authors declare no conflicts of interests.

## ETHICS STATEMENT

The studies have been approved by the appropriate institutional research ethics committee and have been performed in accordance with the ethical standards as laid down in the 1964 Declaration of Helsinki and its later amendments or comparable ethical standards. The BRCF was established to obtain human biospecimens from patients undergoing treatment at KUMC as part of a protocol approved by their internal Human Subjects Committee (HSC #5929).

## Supporting information


Figure S1.

Figure S2.

Table S1.

Table S2.

## Data Availability

The mitochondrial sequencing datasets from all the subjects are available in the NCBI‐SRA repository (#PRJNA907028). This Sequence Read Archive (SRA) submission will be released on December 21, 2023 and available from https://www.ncbi.nlm.nih.gov/sra/PRJNA907028.
