# Supplementary figures and images for: Profiling mitochondrial DNA mutations in tumors and circulating extracellular vesicles of triple‐negative breast cancer patients for potential biomarker development
Source: FASEB Bioadv. 2023 Sep 8;5(10):412–26. doi: 10.1096/fba.2023-00070 (PMC10551276; doi:10.1096/fba.2023-00070)

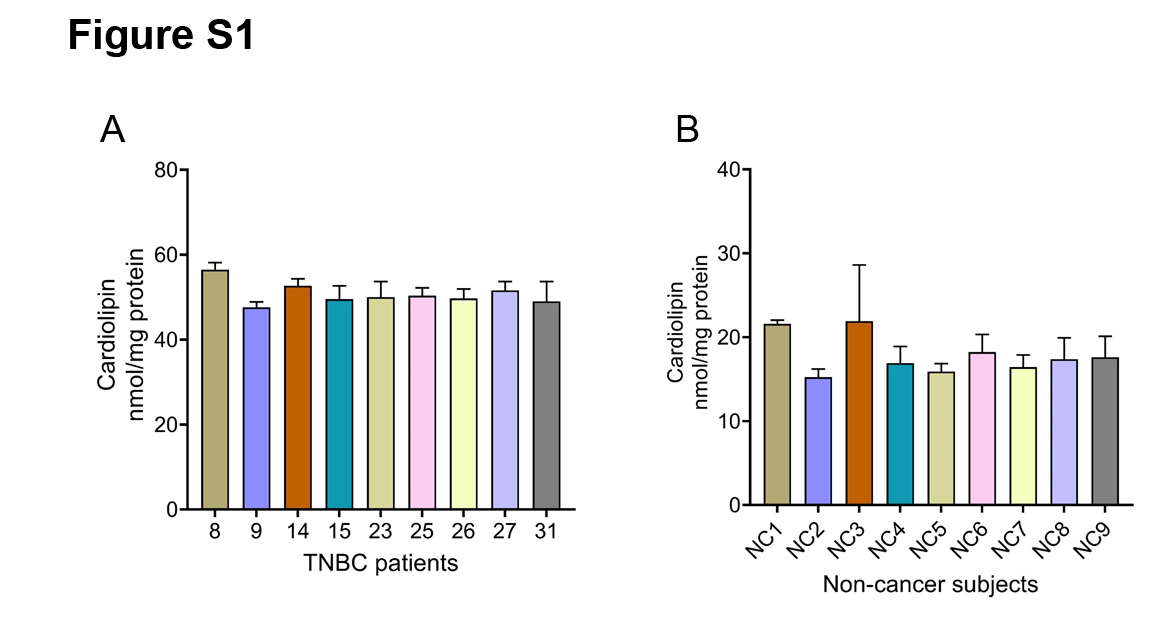

Supplement: Supplementary file 1 — Figure S1. [file FBA2-5-412-s001.tif]

Figure S2

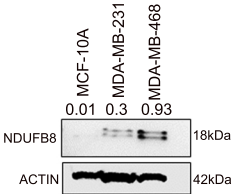

Supplement: Supplementary file 2 — Figure S2. [file FBA2-5-412-s002.pdf]
