# Supplementary material for: Profiling mitochondrial DNA mutations in tumors and circulating extracellular vesicles of triple‐negative breast cancer patients for potential biomarker development
Source: FASEB Bioadv. 2023 Sep 8;5(10):412–26. doi: 10.1096/fba.2023-00070 (PMC10551276; doi:10.1096/fba.2023-00070)
Supplement: Supplementary file 3 — Table S1. [file FBA2-5-412-s004.docx]

**Table S1.** Demographic features of the TNBC patients.

| **ID** | **Age** | **Type of sample^1^** | **Ancestry^2^** | **Stage** | **Grade** | **Diagnosis** | **Serum^3^** |
| --- | --- | --- | --- | --- | --- | --- | --- |
| 1 | 55 | FFPE | Europe | II | II | Invasive ductal carcinoma with squamous features | No |
| 2 | 84 | FFPE | Europe | II | III | Invasive ductal carcinoma | No |
| 3 | 52 | FFPE | *Africa* | II | III | Invasive ductal carcinoma | No |
| 4 | 51 | FFPE | Europe | IV | III | Invasive ductal carcinoma | No |
| 5 | 53 | FFPE | Europe | II | III | Invasive ductal carcinoma | No |
| 6 | 36 | FFPE | Europe | II | III | Invasive ductal carcinoma | No |
| 7 | 49 | FFPE | Europe | I | III | Invasive ductal carcinoma | No |
| 8 | 53 | FFPE | Europe | II | III | Invasive ductal carcinoma | **Yes** |
| 9 | 53 | FFPE | Europe | II | III | Invasive ductal carcinoma | **Yes** |
| 10 | 76 | FFPE | Europe | II | III | Invasive ductal carcinoma | No |
| 11 | 48 | FFPE | Europe | II | III | Invasive ductal carcinoma | No |
| 12 | 53 | FFPE | Europe | II | III | Invasive ductal carcinoma | No |
| 13 | 85 | FFPE | Europe | I | III | Invasive ductal carcinoma | No |
| 14 | 66 | FFPE | Europe | II | III | Invasive ductal carcinoma | **Yes** |
| 15 | 38 | FFPE | *Africa* | I | III | Invasive ductal carcinoma | **Yes** |
| 16 | 60 | FFPE | Europe | II | III | Invasive ductal carcinoma | No |
| 17 | 61 | FFPE | Europe | I | III | Invasive ductal carcinoma | No |
| 18 | 81 | FFPE | *Africa* | II | III | Invasive ductal carcinoma | No |
| 19 | 50 | FFPE | Europe | II | III | Invasive ductal carcinoma | No |
| 20 | 71 | FFPE | Europe | III | III | Invasive ductal carcinoma | No |
| 21 | 86 | FFPE | Europe | III | III | Invasive ductal carcinoma with apocrine features | No |
| 22 | 73 | Frozen | Europe | III | III | Invasive ductal carcinoma | No |
| 23 | 57 | Frozen | Europe | II | II | Adenoid cystic carcinoma | **Yes** |
| 24 | 64 | Frozen | Europe | I | I | Fibromatosis like metaplastic carcinoma | No |
| 25 | 84 | Frozen | Europe | II | III | Invasive ductal carcinoma | **Yes** |
| 26 | 60 | Frozen | Europe | II | III | Invasive ductal carcinoma | **Yes** |
| 27 | 40 | Frozen | *Africa* | II | III | Invasive ductal carcinoma | **Yes** |
| 28 | 74 | Frozen | Europe | I | III | Invasive ductal carcinoma | No |
| 29 | 56 | Frozen | *Africa* | I | III | Invasive ductal carcinoma | No |
| 30 | 62 | Frozen | *Africa* | III | III | Invasive ductal carcinoma | No |
| 31 | 48 | Frozen | Europe | I | III | Invasive ductal carcinoma | **Yes** |
| 32 | 55 | Frozen | *Africa* | III | III | Invasive ductal carcinoma | No |

^1^FFPE: Formalin-fixed paraffin embedded; ^2^Ancestry: Determined using Haplogrep 2.4.0; ^3^Serum: Yes- serum sample available, No- Serum sample not available.

-
